# Pectoral Dimorphism Is a Pervasive Feature of Skate Diversity and Offers Insight into their Evolution

**DOI:** 10.1093/iob/obz012

**Published:** 2019-06-15

**Authors:** C M Martinez, B H Kao, J S Sparks, P C Wainwright

**Affiliations:** 1 Department of Evolution and Ecology, University of California, Davis, CA 95616, USA; 2 Department of Aquaculture, National Taiwan Ocean University, Keelung City 20224, Taiwan; 3 Department of Ichthyology, Division of Vertebrate Zoology, American Museum of Natural History, New York, NY 10024, USA

## Abstract

Mature skates (Batoidea: Rajoidei) display a unique form of sexual dimorphism in which males develop a concave anterior pectoral fin, giving them a bell-shaped appearance. Recent work has linked the male-specific transformation to differential skeletal development that is coincident with the rapid elongation of claspers, cartilage-supported intromittent organs. Still, little is known about the prevalence of pectoral dimorphism across skates or of interspecific variation in its expression. Here, we use various morphological approaches to broadly explore pectoral dimorphism in skates, with the goal of understanding its significance in their evolutionary history. We find that pectoral fin sexual dimorphism exists across skate diversity, positively identifying its presence in at least 131 species spanning 33 genera, approximately 40% of valid species. Further, we show that the nature of male–female shape change is largely consistent across species, but that it differs in its magnitude at a biologically meaningful scale. Finally, we use the pygmy skate *Fenestraja plutonia* as a case study to illustrate ontogenetic patterns in the development of pectoral fin dimorphism, additionally identifying sex-based differences in the pelvic girdle and jaw. Our work suggests that the diversity of pectoral dimorphism in skates is linked to comparative growth and maturation, and potentially to processes underlying reproductive and life history diversification within the group.

## Synopsis

El Dimorfismo Pectoral es una Característica Generalizada de la Diversidad de Patines y Ofrece una Perspectiva de su Evolución Los patines (Batoidea: Rajoidei) muestran una forma única de dimorfismo sexual en el que los machos desarrollan una aleta pectoral anterior cóncava que les da una apariencia de campana. Estudios recientes han relacionado este dimorfismo en los machos con el desarrollo esquelético diferencial que coincide con la rápida elongación de los gonopterigios, órganos intromitentes soportados por cartílago. Sin embargo, poco se sabe acerca de la prevalencia del dimorfismo pectoral en patines o de la variación interespecífica en su expresión. En este estudio abordamos varios enfoques morfológicos para explorar ampliamente el dimorfismo pectoral en patines, con el objetivo de comprender su importancia en su historia evolutiva. Identificamos dimorfismo sexual en al menos 131 especies que abarcan 33 géneros, aproximadamente el 40% de las especies válidas. Además, mostramos que la naturaleza del cambio de forma masculino–femenino es en gran medida consistente en todas las especies, pero que difiere en su magnitud en una escala biológicamente significativa. Por último, utilizamos el patín pigmeo *Fenestraja plutonia* como estudio de caso para ilustrar los patrones ontogenéticos en el desarrollo del dimorfismo de la aleta pectoral, además de identificar dimorfismo sexual a nivel de cintura pélvica y mandíbula. Nuestro trabajo sugiere que la diversidad del dimorfismo pectoral en los patines está relacionada con el crecimiento y la maduración comparativos y, potencialmente, con los procesos subyacentes a la diversificación de la reproducción e historias de la vida dentro del grupo. Translated to Spanish by S. Hinojosa (hinojosa.silvia@gmail.com)

O Dimorfismo Peitoral é uma Característica Difusa da Diversidade do Skate e Oferece Informações sobre sua Evolução Rajídeos maduros (Batoidea: Rajoidei) exibem uma forma única de dimorfismo sexual em que os machos desenvolvem uma nadadeira peitoral anterior côncava, dando-lhes uma aparência em forma de sino. Trabalhos recentes correlacionam a forma específíca dos machos ao desenvolvimento diferencial do esqueleto, que coincide com o rápido alongamento dos clásperes, órgãos intromitantes cartilaginosos. Entretanto, pouco se sabe sobre a prevalência do dimorfismo peitoral em rajideos, ou sobre a variação interespecífica dessa expressão. Nesse trabalho utilizamos diversas abordagens morfológicas para explorar de forma ampla o dimorfismo peitoral dessas raias, com o objetivo de compreender sua significancia em relação à história evolutiva desse grupo. Descobrimos que o dimorfismo sexual da nadadeira peitoral existe entre de toda a diversidade dos rajídeos, observando sua presença em ao menos 131 espécies espalhadas por 33 gêneros, compreendendo aproximadamente 40% das espécies válidas. Além disso, mostramos que a natureza da mudança da forma entre machos e fêmeas é consistente entre as espécies, mas que diferem em sua magnitude em uma escala biologicamente significativa. Finalmente, usamos o rajideo pigmeu *Fenestraja plutonia* como um estudo de caso para ilustrar padrões ontogenéticos no desenvolvimento do dimorfismo da nadadeira peitoral, além de identificar diferenças baseadas no sexo na cintura pélvica e na mandíbula. Nosso trabalho sugere que a diversidade do dimorfismo peitoral em rajídeos está ligada ao crescimento e maturação comparativos e, potencialmente, aos processos subjacentes à diversificação reprodutiva e de história de vida dentro do grupo. Translated to Portuguese by J.P. Fontenelle (jp.fontenelle@mail.utoronto.ca)

Brustflossen-Dimorphismus Ist ein weit Verbreitetes Merkmal der Diversität der Rochen und bietet Einblick in ihre Evolution (Pectoral Dimorphism is a Pervasive Feature of Skate Diversity and Offers Insight into their Evolution) Vollentwickelte Rochen (Batoidea: Rajoidei) zeigen eine einzigartige Form von Sexualdimorphismus, bei der Männchen eine konkave anteriore Brustflosse entwickeln, die ihnen eine glockenförmige Erscheinung verleiht. Jüngste Arbeiten haben die spezifisch männliche Transformation mit differentieller Skelettentwicklung in Verbindung gebracht, die mit der raschen Dehnung der Klaspern, knorpelgestützter Begattungsorgane, zusammenfällt. Noch ist wenig bekannt über die Prävalenz des Brustflossen-Dimorphismus bei Rochen oder über die interspezifische Variation in seiner Ausprägung. Hier verwenden wir verschiedene morphologische Ansätze, um den Brustflossen-Dimorphismus in Rochen umfassend zu erforschen, mit dem Ziel dessen Bedeutung in ihrer Evolutionsgeschichte zu verstehen. Wir stellen fest, dass der Sexualdimorphismus der Brustflosse über die gesamte Vielfalt der Rochen hinweg existiert, und können seine Präsenz in mindestens 131 Arten aus 33 Gattungen, etwa 40% der gültigen Arten, positiv identifizieren. Des Weiteren zeigen wir, dass die Gestalt der männlich–weiblichen Formänderung weitgehend artübergreifend ist, sich aber in ihrer Größenordnung auf biologisch bedeutsamer Ebene unterscheidet. Schließlich verwenden wir den Rochen *Fenestraja plutonia* als Fallstudie, um ontogenetische Muster bei der Entwicklung des Brustflossen-Dimorphismus zu veranschaulichen und zusätzlich geschlechtsspezifische Unterschiede im Beckengürtel und im Kiefer zu identifizieren. Unsere Arbeit legt nahe, dass die Diversität des Brustflossen-Dimorphismus in Rochen mit komparativem Wachstum und Reifwerden in Verbindung steht, sowie möglicherweise mit Prozessen, die der Diversifizierung der Reproduktions- und Lebensgeschichte innerhalb der Gruppe zugrunde liegen. Translated to German by F. Klimm (frederike.klimm@biologie.uni-freiburg.de)

## Introduction

Sexual dimorphism represents an important, albeit sometimes overlooked, source of intraspecific variation, upon which both sexual and natural selection may act (Lande 1980). Evidence from theoretical modeling (Rice 1984; Bolnick and Doebeli 2003) and empirical studies (Vitt and Cooper 1985; Masta and Maddison 2002; Butler et al. 2007) suggests that dimorphism can be indicative of, or directly involved in, processes underlying evolutionary diversification. The implications of morphological divergence based on sex are all the more consequential when the traits involved are functionally important (Shaffer et al 2001; McGee and Wainwright 2013) or otherwise vital to resource partitioning (Schoener 1967; Shine 1989). Given the potential for dimorphic trait variation to influence selection, it follows that this variation can provide context and insight for macroevolutionary patterns.

Historically, the most common examples of taxonomically widespread sexual dimorphism involve body size variation. Size dimorphism has been hypothesized to result from a number of factors, including competitive release between males and females and differences in metabolic needs in relation to parental care (Moors 1980; Cheverud et al. 1985; Badyaev 2002). Comparative evaluations of shape dimorphism are fewer in number, but they emphasize how sex-specific differentiation of morphological features can serve as an integral component to larger patterns of diversity (Dunn et al. 2001; Sanger et al. 2013; Kaliontzopoulou et al. 2015). Here, we describe sexual dimorphism in the pectoral fin shape of skates (Batoidea: Rajoidei) and discuss its relevance to their diversity and evolution.

Skates are a diverse group of fishes, comprising approximately 300 recognized species (Froese and Pauly 2018), distributed among four families (Anacanthobatidae, Arhynchobatidae, Gurgesiellidae, and Rajidae) in the suborder Rajoidei. This makes them the largest subordinal group of extant cartilaginous fishes. A new time-calibrated phylogeny suggests that much of this diversity has accumulated recently and rapidly in comparison to other batoid fishes (Stein et al. 2018). The extraordinary species richness of skates has remained somewhat of a mystery, especially given that many species are thought to be ecologically similar (e.g., Bizzarro et al. 2007). At the same time, research on batoid pectoral shapes suggests that morphological variation in skates could be underestimated, hinting at a greater diversity of lifestyles than is currently known (Martinez et al. 2016a). The presence of dimorphism in such a functionally vital part of the skate body plan, its pectoral fins, makes it a promising resource for improving our understanding of the group’s diversity.

**Fig. 1:**
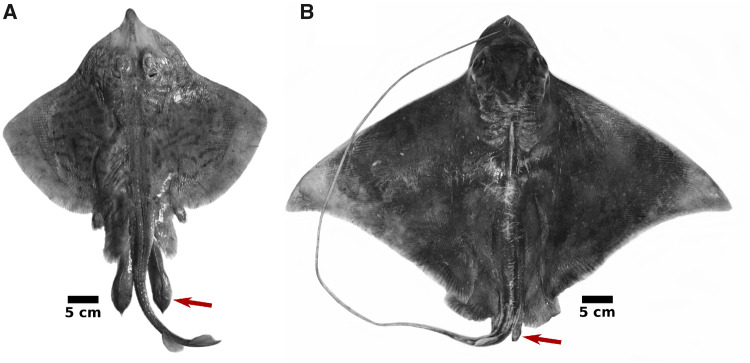
Dorsal photographs of a mature male skate (**A,***Raja eglanteria*) and ray (**B,***Myliobatis freminvillei*), representing relative differences in clasper size and morphology (red arrows).

Sexual dimorphism of skate pectoral fins has been documented in several species (Oddone and Vooren 2004; Ebert et al. 2007; Orlov and Cotton 2011; Martinez et al. 2016b), but its prevalence across Rajoidei has never been formally determined. It is characterized by what has been referred to as a “bell-shaped” disc (Ebert et al. 2007) that develops in sexually mature males, where a distinct concavity appears in the anterior half of the pectoral fin. Although intraspecific differences in pectoral shape between males and females can be quite large, interspecific variation in the magnitude of dimorphism has also been observed (Martinez et al. 2016b). There is currently no evidence that pectoral dimorphism is associated with ecological factors like sex-specific habitat utilization or differences in mobility. Rather, previous work suggests that it is caused by the development of claspers (Martinez et al. 2016b), paired male intromittent organs (Fig. 1). The link between the two, while not initially obvious, is believed to involve a currently unidentified endocrinological pathway that promotes rapid elongation of clasper cartilages during sexual maturation, but which also causes incidental growth and differential development of other skeletal features (Kajiura et al. 2005), including those supporting the pectoral fins. There is also limited evidence that species with faster rates of sexual maturation and clasper elongation, but not necessarily greater overall clasper size, have more intense pectoral dimorphism (Martinez et al. 2016b). One idea for the mechanism underlying this relationship is that rapid clasper growth, and other correlated skeletal changes, occur over a smaller window of overall somatic growth such that the body cannot accommodate (i.e., grow into) these changes compared with species in which development is spread over a longer growing period. Additionally, observed relationships between skate claspers and pectoral fins are consistent with previous work on developmental pathways of cephalic lobes in pelagic rays, which suggests an intriguing shared pattern of gene expression between claspers and the anterior pectoral fins of batoids (Swenson et al. 2018). Additionally, in the bonnethead shark, *Sphyrna tiburo*, clasper development is associated with changes in rostrum morphology, leading to divergence in male and female head shapes (Kajiura et al. 2005). The proposed clasper–fin relationship may also have macroevolutionary implications, as there is good reason to believe that clasper morphology and development have played a major role in skate diversification.

Claspers evolved from modifications to pelvic fins and are considered one of the primary synapomorphies characterizing chondrichthyan fishes (Lund and Grogan 1997; Grogan and Lund 2004). However, there is considerable variation in the relative size and morphology of the organs across clades. Two primary clasper morphotypes have been identified in batoids (McEachran and Aschliman 2004); an elongate form that is depressed in shape distally and is found primarily in skates and guitarfishes, and a shortened and more cylindrical form that occurs mostly in rays from the Order Myliobatiformes and in torpedo rays (Fig. 1). In skates, clasper morphologies are particularly diverse, to an extent that they have been described as “outstandingly significant in the distinction of species” (Hubbs and Ishiyama 1968). Owing to its relationship with clasper development, pectoral dimorphism may therefore be tied to processes underlying the diversity of reproductive or life history strategies in skates.

In this study, we integrate multiple lines of morphological evidence to explore patterns of sexual dimorphism in skates, both at a broad comparative scale and in further depth within a single species. First, we used geometric morphometrics to characterize the nature and consistency of pectoral dimorphism across skate genera. Given the proposed presence of a common mechanism for pectoral dimorphism (i.e., clasper-correlated skeletal growth), we predicted that the manner of male-to-female shape change would be largely consistent across skates, but that it would vary in magnitude. We also tested the extent to which male clasper lengths explained variation in the magnitude of dimorphism across skates. We predicted a weak relationship that would exclude overall clasper size as the driver for dimorphic diversity, leaving open the possibility of clasper growth rate as the primary factor. Using dimorphic features identified with geometric morphometrics in this study and in Martinez et al. (2016b) as a guide, we also undertook a broad survey of the literature to document the ubiquity of pectoral fin dimorphism as a feature of skate diversity. Despite having only been identified in a small number of skates, the presence of dimorphism in distantly related species led us to predict that it would be found across the suborder Rajoidei. Finally, we assembled ontogenetic series for external and skeletal morphologies in male and female *Fenestraja plutonia*, a species of pygmy skate in which males possess claspers of lengths up to 49% of their disc width. We used this system as a case study for the development of dimorphism. Based on the proposed clasper-driven mechanism, we predicted that the magnitude of dimorphism would be greater at adult stages and that males would differ more than females from their juvenile forms. We also took advantage of the opportunity to identify other potential forms of dimorphism within the endoskeleton.

## Materials and methods

### Form and consistency of pectoral dimorphism

We used geometric morphometrics to evaluate pectoral dimorphism and assess the relative consistency of associated shape changes across skates. Images of two specimens (one male and one female) were chosen to represent each species and were gathered from several sources that included published photographs from the primary literature, specimens analyzed in Martinez et al. (2016a), and images from fishbase.org (Froese and Pauly 2018). Sampling included representative species from 21 genera of skate as well as the guitarfish *Zapteryx brevirostris* (Rhinobatidae; Supplementary Table S1). We took care in choosing images where pectoral fins accurately represented adult pectoral morphologies for the species in question. We used tpsDIG2 (Rohlf 2015) to digitize 35 landmarks along the outer margin of one pectoral fin, including 33 sliding semi-landmarks, plus two fixed landmarks flanking the ends, following methods of Martinez et al. (2016b) (Supplementary Fig. S1). Endpoints of curves were the anterior extent of the propterygium and the posterior insertion of the pectoral fin onto the body, near the distal end of the metapterygium. We chose the pectoral fin that was in the best physical condition, reflecting any left-side fins along their bilateral axis so that all fin margins faced to the right. Shape alignment was done in the package *geomorph* (Adams and Otarola-Castillo 2013) in R (R Core Team 2018).

We were interested in assessing the relative consistency in the nature of male-to-female shape change across skate species and variation in the magnitude of shape dimorphism. In geometric morphometrics, shape change between two states (here, adult male and female pectoral fins) is typically evaluated by comparison of shape vectors, with endpoints at the average shape for each state (e.g., Collyer and Adams 2007). Given the sampling design of this study, lacking intraspecific variation from which to estimate mean shapes, we generated vectors with endpoints at the two specimens chosen to represent a species. As a consequence, we could not statistically test pairwise comparisons of vector traits (their lengths and angular displacements) between all sets of species. Rather, we present the collective diversity of vector lengths to illustrate the range of dimorphic magnitudes intrinsic to skates. Vector lengths, or Procrustes distances, were calculated as the square root of the sum of squared differences between landmark coordinates for corresponding female–male species pairs. To assess collective patterns of vector angles, which signify differences in the direction or manner of shape change, we measured their displacements relative to a common baseline, shape variation along the second principal component (PC 2) from a principal component analysis (PCA) of pectoral shapes. To do this, we used the “plotTangentSpace” function in *geomorph* and obtained shape coordinates corresponding with the minimum and maximum PC 2 scores. These shapes were used to create the baseline shape-change vector for comparisons against dimorphism shape vectors (i.e., estimates were made in the full dimensionality of pectoral shape data). Shapes along PC 2 were chosen due to the relatively consistent differentiation of male versus female forms along this axis. Angular displacements between shape vectors and PC 2 were calculated, following the methods outlined in Collyer and Adams (2007). Prior to angle calculations, all vectors were divided by their respective lengths and a PCA was done on the resulting scaled vectors to visualize variation in dimorphic shape changes (e.g., Zelditch et al. 2016).

Even with careful specimen selection, we acknowledge the inherent limitations of our sampling scheme for robust statistical comparisons. However, we rely on observations repeated across 21 skate genera in order to recover preliminary observations of overriding patterns of shape variation associated with pectoral dimorphism. We visualized relative diversity of vector traits with a phylogenetic bubble plot, using the “dotTree” function in the R package *phytools* (Revell 2012). Phylogenetic relationships shown herein were based on a distribution of 1000 trees of all batoids that was obtained from an online supplement to Stein et al. (2018). A maximum clade credibility tree was generated in TreeAnnotator version 1.8.4 (Drummond et al. 2012; Rambaut and Drummond 2016) and then trimmed for the species of interest.

Finally, we used phylogenetic generalized least-squares regression (PGLS) to test whether the magnitude of sexual dimorphism was related to the lengths of male claspers. For this, clasper lengths were measured from the same dorsal images of males that pectoral shapes were obtained. We measured claspers from the posterior insertion of the pelvic fin on the body (i.e., the most proximal part of the clasper visible from a dorsal view) to the distal end of the clasper. When both claspers were fully visible, we took the average, but some were based on a single clasper. We did not include males from *Leucoraja* or *Zapteryx* in the PGLS, as neither clasper was visible in the images used. Before the regression was performed, clasper lengths were standardized by taking residuals of a regression of log-clasper length on log-body size (i.e., centroid size of associated pectoral fin).

### Prevalence of dimorphism in skates

In order to evaluate how widespread pectoral dimorphism is in skates, we undertook a formal survey of species diversity using images from various sources (*n *=* *70 references; Supplementary Table S1 and Supplementary Document S1). Conveniently, the wide availability of pectoral fin images from rajoid species descriptions and research papers offers a valuable source of information that can be gathered for a large number of skates. We made use of this much-expanded taxonomic sampling of skate images, some of which were not suitable for quantitative analyses (e.g., not taken in standardized position or lacking an adequate scale), but nonetheless displayed morphological differences between males and females. Therefore, dimorphism was determined qualitatively from visual inspection of images, but the assessment itself was grounded in the detection of dimorphic features of pectoral fins that had been quantitatively identified elsewhere in this study and in Martinez et al. (2016b).

We preferentially used photos to make assessments, but for limited occasions we also used illustrations from some older species descriptions; the type of source image was noted. For confirmation of dimorphism, there needed to be at least one image of a mature male and one of a mature female. In some species, only images of males were found, but they possessed characteristic dimorphic features in their pectoral morphologies. We listed these as provisionally dimorphic. Although dimorphism ranges from strong to very weak, we only documented its affirmative presence in a binary manner. This conservative approach was taken in order to eliminate an incorrect designation of no dimorphism, when in fact it does exist for the species in question, but that it was simply not clear from the images assessed.

### Dimorphism in *F. plutonia*; a case study

In order to provide context to broader patterns of sexual dimorphism across species of skate, we also focused on more detailed evaluation of ontogenetic variation within a single species, *F.plutonia*. To do this, we examined the external pectoral shape and endoskeleton morphology of *F. plutonia* with geometric morphometrics and mensural data, respectively. In total, we evaluated 43 individuals, including 21 males and 22 females that ranged from juvenile (50.6 mm, minimum disc width) to sexually mature (109.5 mm, maximum disc width) life stages (Supplementary Table S2). Ethanol-preserved specimens came from collections held at the American Museum of Natural History and the Harvard Museum of Comparative Zoology. We took photos of specimens from a dorsal view, with pectoral fins lying flat.

**Fig. 2:**
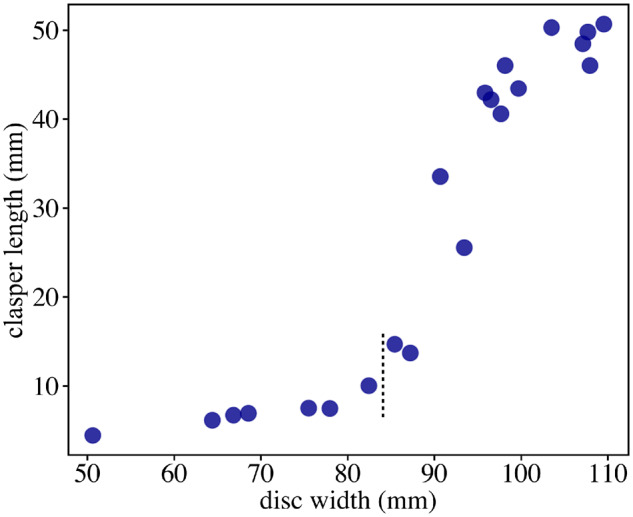
Average clasper lengths per individual plotted against disc width in male *Fenestraja plutonia*. The onset of sexual maturation is shown by a vertical dashed line segment that demarcates an inflection point after which clasper length rapidly increases.

For comparisons of external morphology within *F. plutonia*, we again characterized pectoral dimorphism as vectors of shape change. However, unlike the interspecific vectors computed previously, we now had sufficient sampling to statistically test for differences in vector traits (i.e., their magnitudes and angles). We replicated the landmark placement described in the previous section (33 sliding semi-landmarks and 2 fixed landmarks) to capture the curvature of the outer fin margin. As before, digitizing was done in tpsDIG2 and shape alignment was achieved in *geomorph*. Maturity was determined based on the development of male claspers. In skates, claspers undergo rapid elongation at the onset of sexual maturity (Sosebee 2005), and in species like *F. plutonia*, the approximate body size at which this transition takes place can easily be identified by plotting clasper length against disc width (Fig. 2). In our dataset, there was a clear and rapid increase in clasper length for individuals with disc widths >84 mm. We used this value as a maturity threshold, below which individuals were classified as immature and would therefore not have experienced the level of clasper growth needed to impact their pectoral morphologies. Specimens >84 mm included both maturing and fully mature individuals (“mature” hereafter, for simplicity), where we expect clasper elongation to have influenced pectoral shapes to some degree. We used the same size cutoff for females, as diagnostics for their maturation state, like egg development and cloaca depth, were not found in preserved specimens. Finally, we did two sets of analyses on shape vectors, the first comparing ontogenetic shape change (immature to mature) within each sex and second, comparing sexually dimorphic shape change (male to female) at comparable maturation stages. These analyses were done with the “trajectory.analysis” function in *geomorph* and were based on a 10,000-permutation procedure.

**Fig. 3:**
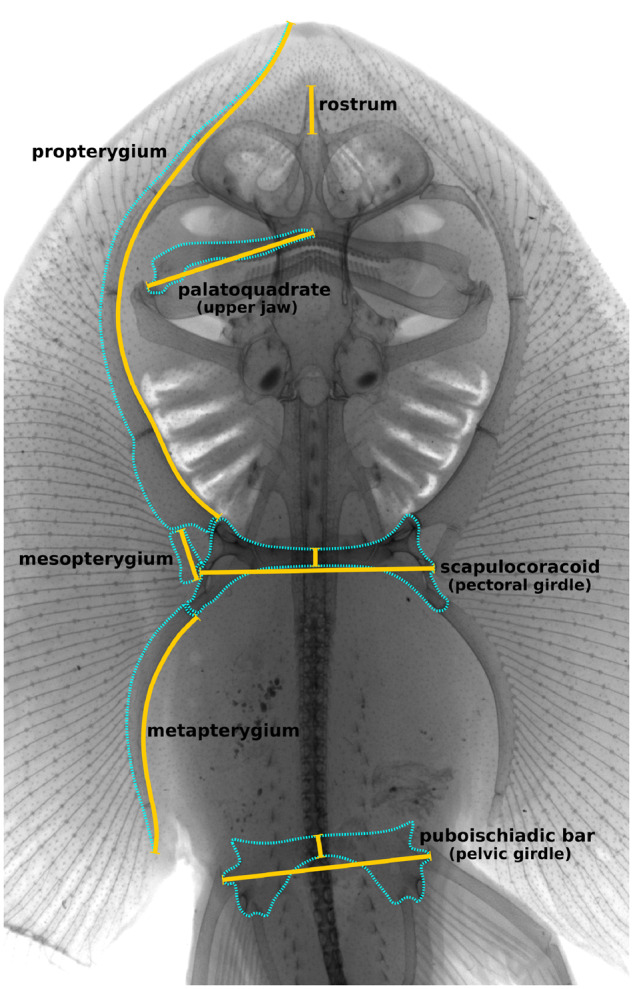
Measurements made on endoskeletal traits in *F. plutonia* (yellow lines).

In the same specimens evaluated for external pectoral fin shape, we took X-rays and made linear measurements of several endoskeletal traits (Fig. 3). Measurements included, 1) the length of the puboischiadic bar (pelvic girdle) at its joint with the first radial of the crura or elongated anterior pelvic fin lobe, 2) the width of the puboischiadic bar along the axis of bilateral symmetry, 3) the length of the palatoquadrate (upper jaw), 4) length of the scapulocoracoid (pectoral girdle) at its condyles connecting to the mesopterygia, 5) the width of the scapulocoracoid along the axis of bilateral symmetry, 6) the length of the rostrum as it projects beyond the neurocranium, 7) the length of the propterygium (anterior basal cartilage of pectoral fin) along its medial boundary, 8) the length of the mesopterygium (middle basal pectoral cartilage) along its largest axis, and 9) the length of the metapterygium (posterior basal pectoral cartilage) along its medial boundary. We used ANCOVAs to test whether endoskeletal features of males and females differed in their allometric slopes relative to the natural logarithm of disc width, and if not, whether these features differed in length with log-disc width as the covariate. Finally, in order to supplement the visualization of some of the morphological differences in skeletal traits, we used micro-CT scans of two adult *F. plutonia* (AMNH 76564, mature female, DW = 108.03 mm; and AMNH 76564, mature male, DW = 107.94 mm).

## Results

### Form and consistency of pectoral dimorphism

**Fig. 4:**
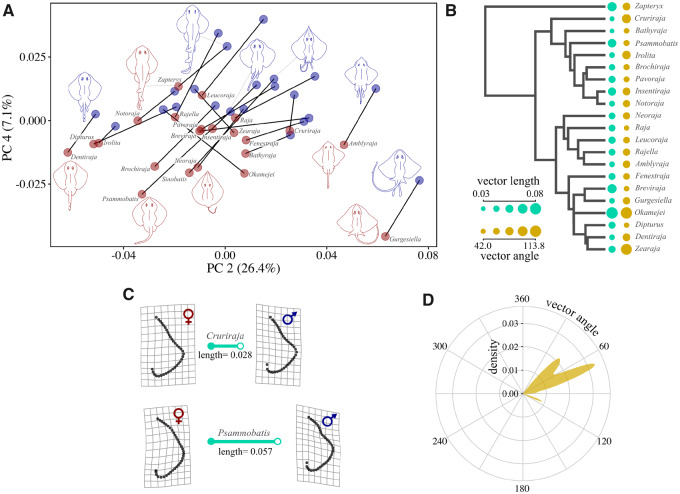
(**A**) Pectoral shape variation in male–female pairs for 21 genera of skate and one guitarfish (*Zapteryx*), each connected by a dimorphism vector (black lines). PCs 2 and 4 are shown, as these axes most consistently differentiated shape change between females (red) and males (blue). (**B**) Dimorphism vector traits are plotted as dots, with values scaled by size, next to a phylogenetic tree of the study species. Vector length (teal) is the magnitude of dimorphism or Procrustes distance, and angle (dark yellow) is the difference in vector orientation relative to a common baseline, in degrees. Vector angles were computed in full shape space from each male–female pair of pectoral shapes to a vector represented by the coordinates of minimum and maximum shapes along PC 2. Note that a dimorphic outlier, *Sinobatis*, is removed from the phylogeny for visualization, as its vector magnitude obscures variation in other species. (**C**) Deformation grids of male–female shape change for select skate species show what a two-fold difference in dimorphic magnitude looks like. Idealized vectors are included in teal and are scaled to relative length. (**D**) A polar density plot is presented for dimorphic vector angles relative to the shape vector along PC 2. The narrow distribution of angles (out of the possible 360 degrees) indicates a similar nature of dimorphic shape change in most skates.

The primary axis of pectoral fin variation in skates (PC 1) was related to interspecific differences in shape, which is not surprising given that the dataset contains species distributed across four rajoid families. However, PCs 2 and 4 consistently differentiated male–female pairs across species (Fig. 4A andSupplementary Fig. S2). PC 2 represented 26.2% of shape variation and males possessed higher scores on this axis for representatives of all genera but two, *Okamejei* and *Zearaja*. In these cases, females had a more anterior pectoral fin apex than males, which was the opposite pattern for other evaluated genera (Supplementary Fig. S3). On PC 4 (7.1% of pectoral shape variation) males had greater scores in every genus. Additionally, angles of female–male shape change vectors to PC2 revealed that in comparison to the full range of possible angular displacements (360°), vectors displayed a total range of 71.7° (Fig. 4B, D). This means that dimorphic changes occupy a small to modest subset of directions through shape space and suggest a level of consistency in the manner of shape change across species.

Relative to females, males possessed convex anterior pectoral fins that resulted in a more prominent, posteriorly oriented fin apex, and more strongly contoured overall appearance (Fig. 4A, C). Additionally, there was often relative expansion of male pectoral fins near the posterior insertion on the body, giving this region a slightly more rounded and lobed shape compared with females. We also recovered the same form of pectoral dimorphism in the guitarfish *Zapteryx*, confirming its existence in batoids beyond skates. Additionally, the magnitude of pectoral dimorphism, or length of female–male shape vectors, was variable (Fig. 4B, C). For example, the genus with the strongest dimorphism, *Sinobatis*, displayed shape divergence over five times that of the weakest, *Bathyraja*. Even without *Sinobatis*, which was an outlier in the strength of dimorphism, the difference was over three-fold. In *Sinobatis*, males possessed the same characteristic anterior concavity seen in other genera, but the posterior region of their pectoral fin was well-differentiated from the female form; females had minimal posterior extension of the fin beyond its attachment to the body, while male fins extended well beyond this point. It should be reiterated that each genus was represented by a single species, so we suggest caution with interpretations beyond the presence of variation across taxa. Lastly, we found a weak and statistically non-significant relationship between the magnitude of pectoral dimorphism and clasper length (PGLS; *F*_1,18_ = 1.10, *R*^2^ = 0.057, *P *=* *0.31), and it was even weaker with the dimorphic outlier, *Sinobatis*, removed (*F*_1,17_=2.23× 10^−6^, *R*^2^=1.3×10^−7^, *P *=* *0.99) (Supplementary Fig. S4).

### Prevalence of dimorphism in skates

In total, we identified sexual dimorphism in 131 species of skate, from 33 of 35 recognized genera, finding it to be present in all species that we could obtain images for. We were not able to assess dimorphism in *Dactylobatus* or *Pseudoraja* due to a lack of sufficient images. We determined that 17 additional species were provisionally dimorphic, as only images of males with characteristic pectoral features were available for viewing. These results suggest that some level of pectoral dimorphism, as defined above, is present across rajoid diversity. The additional presence of dimorphism in *Zapteryx*, again, provides evidence of this pattern existing in other batoid taxa.

### Dimorphism in *F. plutonia*

**Fig. 5:**
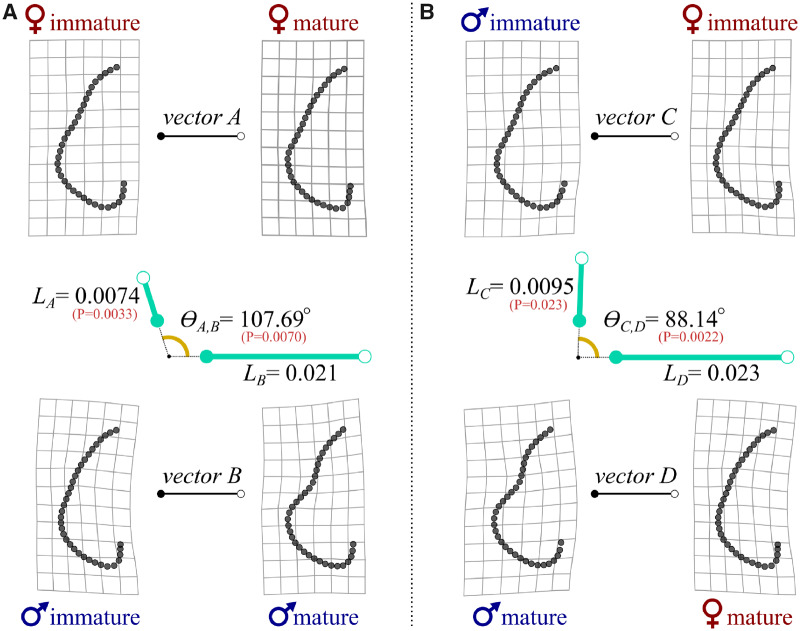
Comparisons of shape change vectors in the species *F. plutonia*. (**A**) Deformation grids for ontogenetic shape change between sexually immature versus mature females (top) and males (bottom). In the center, an idealized diagram shows corresponding comparisons of vector lengths, *L* (teal) and the angle between the two vectors, *θ* (dark yellow). *P*-values for vector trait comparisons are provided in red. Note that shape changes between maturation states are greater in males than females. (**B**) A similar set of vector comparisons, in this case for dimorphic shape change between females and males at immature (top) and mature (bottom) stages. The magnitude of sexual dimorphism is greater in mature individuals.

Analyses of shape vectors in *F.plutonia* were used to study within-species patterns of dimorphism. Ontogenetic pectoral shape change from immature to mature stages was nearly three times greater in males than in females (*P *=* *0.0033; Fig. 5A). A statistically significant difference in angle between male and female ontogenetic trajectories (*θ *=* *107.69; *P *=* *0.0022) suggested that the manner of shape change also differed between sexes. Statistical comparison of vectors between sexes at different maturity levels provided information on the nature of dimorphism over ontogeny. Shape dimorphism in *F. plutonia* increased over ontogeny, with mature males and females showing over 2.4 times greater shape change than that observed in immature specimens (*P *=* *0.023; Fig. 5B). Additionally, the angles between vectors were nearly orthogonal (*θ *=* *88.14; *P *=* *0.0022), indicating that the manner of dimorphic shape change was different at the two life stages. At the immature stage, male pectoral fins were slightly more rounded posteriorly than females (Fig. 5B top). In contrast, mature males are much more divergent in shape from females (Fig. 5B bottom), developing the typical suite of shape characteristics seen in other skates (Fig. 4).

**Fig. 6:**
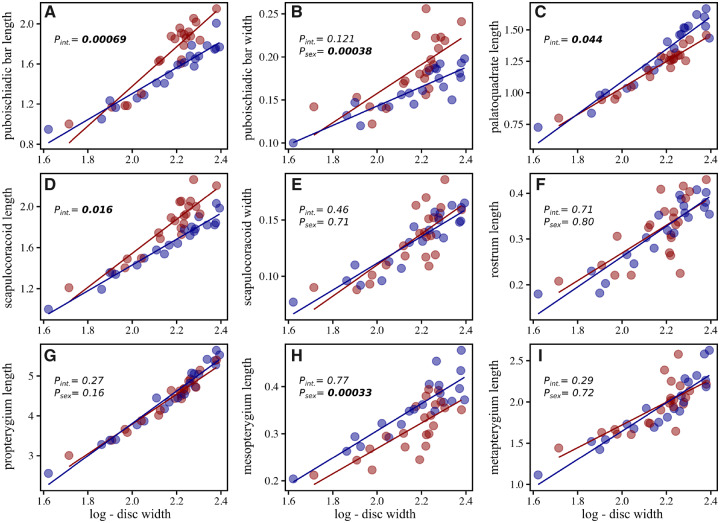
Mensural data taken from radiographs of *F. plutonia*, plotted against log-disc width. Females are shown in red and males in blue. Measured traits include the (**A**) length and (**B**) width of the puboischiadic bar or pelvic girdle, (**C**) length of the palatoquadrate or upper jaw, (**D**) length and (**E**) width of the scapulocoracoid or pectoral girdle, (**F**) length of the rostrum, and lengths of the basal pectoral radials, the (**G**) propterygium, (**H**) mesopterygium, and (**I**) metapterygium. *P*-values from ANCOVAs are also shown, where a significant interaction term (*P*_int._) denotes different slopes between males and females. When the interaction term was not significant, *P*-values from the grouping factor (*P*_sex_) are provided.

**Fig. 7:**
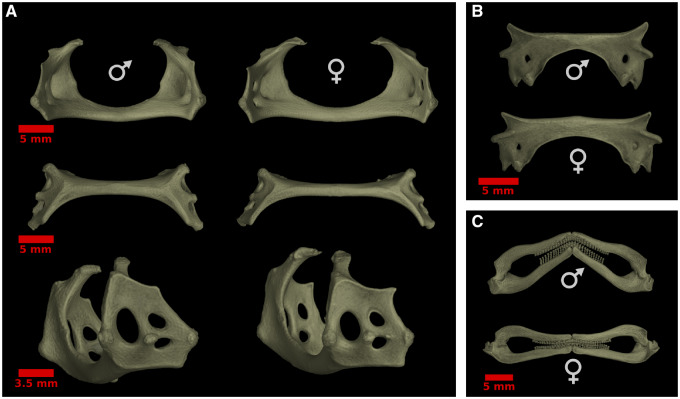
Segmented micro-CT scans of female and male specimens of *F. plutonia* (both from AMNH 76564). (**A**) Pectoral girdles are shown in posterior (top), ventral (middle), and lateral (bottom) views. (**B**) Pelvic girdles and (**C**) jaws are shown in ventral view.

Comparisons of endoskeleton measurements showed that males and females differed significantly in their allometric slopes for puboischiadic bar, palatoquadrate, and scapulocoracoid lengths (Fig. 6A, C, D). In each of these, measurements broadly overlapped at smaller sizes and then diverged in larger individuals. Additionally, while slopes did not differ significantly between sexes, there were statistically significant differences in the width of the puboischiadic bar and the length of the mesopterygium (Fig. 6B, H). Micro-CT scans of male and female *F. plutonia* provided a visualization of the morphological differences that existed in mature individuals. The scapulocoracoid (pectoral girdle) was shorter in mature males, giving it a stout overall appearance (Fig. 7A). The lateral face of the pectoral girdle was also more oblong in males than females, being particularly elongate along the axis containing condyles articulating with basal radials (Fig. 7A, bottom). In comparison, the puboischiadic bar (pelvic girdle) was both longer and wider in mature females (Fig. 7B). Lastly, the palatoquadrate (upper jaw) was greater in length in mature males, and at its most extreme, resulted in a highly angled appearance to the mouth, with the medial symphysis displaced anteriorly relative to similarly sized females (Fig. 7C).

## Discussion

We show that pectoral dimorphism is ubiquitous in skates and that the nature of female–male shape differences is relatively consistent across the group; mature males have fins with an anteriorly concave pectoral margin and a more lobed posterior region. In agreement with our initial predictions, we also found that the magnitude of dimorphism is diverse across species, varying by a factor of five (Fig. 4B). Additionally, detailed morphological examinations of *F.plutonia* revealed that changes in male pectoral fins are associated with sex-based differences in the skeletal development of the pectoral girdle and basal radials that anchor the fin rays (Figs. 5 and 6). This observation supports the findings of previous work by Martinez et al. (2016b) in two species of *Leucoraja* skate. Overall, the pervasiveness of pectoral fin dimorphism in skates (i.e., present in at least 131 species and 33 genera) is striking and suggests that further understanding of its expression will provide valuable context for understanding skate evolution and diversity.

### Drivers of pectoral dimorphism

Rapid elongation of clasper cartilages in male skates, and particularly its effects on the development of other skeletal features, has been proposed as the underlying driver of pectoral fin dimorphism (Martinez et al. 2016b). Consistent with previous work, immature females and males of *F.plutonia* are similar in fin morphologies, but they diverge at maturation due primarily to male shape change (Fig. 5). If clasper growth really is the cause of pectoral dimorphism, we might expect to encounter it more often, as males of all chondrichthyan species possess paired claspers. However, that is not what we observe. For instance, there are no documented cases of pectoral dimorphism of the nature described in this study among myliobatiform rays, which have convergently evolved a similar body plan to skates (Aschliman et al. 2012). As noted previously though, the two groups do differ in clasper size, with skates investing more than rays in the growth of large and morphologically diverse claspers (Fig. 1). For the simple reason that increased somatic growth comes at a metabolic cost (e.g., Wieser 1994), these evolved differences in clasper size presumably require a proportionally larger allocation from the energetic budgets of skates. We therefore suggest that sexual selection for large claspers was an important feature of skate evolution and that observed pectoral dimorphism (also found in the guitarfish *Z.brevirostris*, with similarly large claspers) represents a collateral effect, indicative of the relative investment toward reproduction in these fishes.

While there is growing evidence that the appearance of pectoral dimorphism in skates is related to the presence of elongate clasper cartilages, it is not fully clear what causes the variation in the magnitude of female–male shape differences across species. We found no relationship between clasper length and the magnitude of dimorphism (Supplementary Fig. S4). In fact, some species with comparatively large claspers for their body size display weak dimorphism (e.g., *Gurgesiella dorsalifera*). What then can account for the observed variation in dimorphic magnitude? As mentioned previously, one possibility is variation in the rates at which maturation and clasper elongation occur. A study on clasper growth profiles in seven species of skate showed substantial differences in the rates of clasper growth with body size (Sosebee 2005). This diversity of clasper development underlies important variation in life-history strategies that exists among skates. Larger and longer living species tend to have slower rates of clasper elongation, whereas species that are short-lived, with precocious maturation have much more rapid and well-defined periods of clasper growth (refer to Fig. 3 in Sosebee 2005). In a comparison of *Leucoraja* skates, *Leucorajaerinacea* showed more rapid sexual maturation and clasper elongation and also displayed more intense pectoral fin dimorphism than its slower growing sister species, *L. ocellata* (Martinez et al. 2016b). This work suggested that the larger transformation of male body shape in *L. erinacea* could be caused by the sheer rapidity of skeletal changes in this species relative to overall somatic growth. While robust estimates of life-history traits are not available for the majority of skates, there is evidence of large variation in growth and maturation, even between closely related species (Frisk 2010). Therefore, the diversity of dimorphic magnitude across skates has likely origins in the diversification of life histories, which has loomed large in the evolutionary history of the group.

### Functional implications of sexual dimorphism in skates

In many instances, skate pectoral dimorphism involves major morphological changes in overall male body shape as well as the skeletal architecture that supports the fins (Figs. 4–7; also see Martinez et al. 2016b). It is possible or even likely that these changes carry with them functional implications for swimming performance. For example, in the little skate, *L.erinacea*, swimming kinematics are such that the largest amplitude fin undulations occur at its outer margin, antero-posteriorly centered on its lateral apex (see Figs. 4 and 5 in Di Santo et al. 2017). The bell-shaped morphologies of mature males create a concavity on the fin that in some cases cuts directly into the anterior portion of this region in a manner that must either restrict the area of highest amplitude undulation or displace it posteriorly. Given that skates use their pectoral fins as the primary means of propulsion, with some species undergoing long-distance seasonal movements (Frisk 2010; Frisk et al. 2019), trade-offs between intense dimorphism in rapidly maturing species and migratory behavior may exist. In contrast, this may be less important for species exhibiting greater fidelity to benthic environments, where relatively higher reliance on pelvic fins for small-scale movements could be expected (Koester and Spirito 2003; Macesic and Kajiura 2010).

Given the nature of pectoral dimorphism, an apparently incidental consequence of rapid clasper elongation in males, there was good reason to believe that other skeletal features might show similar patterns of sexual differentiation with maturity. Examination of the endoskeletons of the pygmy skate, *F.plutonia*, indeed revealed additional and biologically important examples of sexual dimorphism. The puboischiadic bar (pelvic girdle) of mature females was both longer and wider than that of males (Fig. 7B). This contrasts the pattern found for the scapulocoracoid (pectoral girdle), where mature females displayed longer, but more gracile structures (Fig. 7A). Further work is needed to evaluate whether differences in pelvic girdle morphology influence the function of pelvic fins during benthic maneuvering, or whether the broader structures of females have implications for their ability to lay wide egg cases. We also observed dimorphism in jaw morphology, where mature males had longer palatoquadrates (upper jaws) than similarly sized females (Fig. 6C). In larger males, the jaw also becomes more acutely angled and anteriorly displaced at its medial symphysis (Fig. 7C). McEachran (1977) noted that this pattern exists in multiple species of skate, describing the mouths of mature males as more “sinuous” in shape. There is no evidence for sex-based dietary differences in skates to suggest that jaw dimorphism influences the partitioning of food resources. Rather, it is likely that differences in jaw morphology are associated with copulatory behavior. Many mature male skates develop sharp, cusped teeth that are used for grasping females during mating (McEachran 1977; Luer and Gilbert 1985) and it is possible that a more anteriorly pointed mouth aides in securing a strong hold onto females. Lastly, we did not find any sign of dimorphism in rostrum length, despite its presence in *L.erinacea* (Martinez et al. 2016b) and the bonnethead shark, *S.tiburo* (Kajiura et al. 2005). One likely explanation for this discrepancy is that *Fenestraja* species possess highly reduced rostral cartilages, compared with most other skates (Fig. 3). Further work will be necessary to evaluate whether these additional cases of dimorphism originate from the same process as pectoral fin differentiation or arise from selection on the specific traits in question.

## Conclusion

Skates are a species-rich group of fishes, but their relatively low ecological and morphological variation have long puzzled researchers. However, the ubiquity of clasper-driven pectoral dimorphism recovered in this study adds a new and intriguing dimension to skate diversity that, until now, has not been fully appreciated. The link between the presence of dimorphism and appearance of large claspers in skates and guitarfishes connects this morphological pattern to an important event in the evolution of batoid reproductive biology. Further work is needed to evaluate the relationship between maturation rates and the magnitude of pectoral dimorphism in skates, but a connection would suggest that dimorphic intensity is indicative of underlying life history variation. This all points to the possibility that the expression of pectoral dimorphism in skates is integrated into the story of their diversification and success, as a group that represents over 44% of batoid species (based on numbers from Froese and Pauly 2018). Future research should also focus on more extensive intraspecific sampling, paired with life history and clasper development data. An additional companion survey of the diversity of pectoral dimorphism in guitarfishes will also provide an interesting comparison to observations made in this study.

## Supplementary Material

obz012_Supplementary_Data
