# Supplementary material for: Pectoral Dimorphism Is a Pervasive Feature of Skate Diversity and Offers Insight into their Evolution
Source: Integr Org Biol. 2019 Jun 15;1(1):obz012. doi: 10.1093/iob/obz012 (PMC7671108; doi:10.1093/iob/obz012)
Supplement: obz012_Supplementary_Data [file obz012_supplementary_data.zip › Document S1.pdf]

Document S1. References used for survey of pectoral fin dimorphism in skates.

[1] Aschliman NC, Ebert DA, Compagno LJV. 2010. A new legskate (Rajoidei: Genus *Cruriraja*) from Southern Africa. Copeia. 3, 364-372.

[2] Bigelow HB, Schroeder WC. 1948. New genera and species of batoid fishes. Journal of Marine Research. 7, 543–566.

[3] Bigelow HB, Schroeder WC. 1962. New and little known batoid fishes from the western Atlantic. Bulletin of the Museum of Comparative Zoology. 128, 159-244.

[4] Bigelow HB, Schroeder WC. 1965. A further account of batoid fishes from the western Atlantic. Bulletin of the Museum of Comparative Zoology. 132, 446-475.

[5] Bottaro M, Consalvo I, Fernando S, Gallus L, Girosi L, Psomadakis PN, Atkinson CJL, Vacchi M. 2008. New records of blonde ray (*Raja brachyura*) from the Ligurian Sea. Marine Biodiversity Records. 1(e72), 1-3.

[6] Capapé C, Guélorget O, Vergne Y, Marquès A, Quignard JP. 2006. Skates and Rays (Chondrichthyes) from waters off the Languedocian coast (Southern France, Northern Mediterranean): a historical survey and present status. Annales: Series Historia Naturalis. 16(2), 165-178.

[7] Fish F. From images contributed to Martinez et al. 2016.

[8] Fisheries and Oceans Canada. Online database: http://www.dfo-mpo.gc.ca/species-especes/skates/identify/index-eng.html. Accessed 2 December 2018.

[9] Froese, Pauly (Eds.). 2018. Fishbase. World Wide Web electronic publication. www.fishbase.org, version (06/2018).

[10] Garrick JAF, Paul LJ. 1974. The taxonomy of New Zealand skates (Suborder Rajoidea), with descriptions of three new species. Journal of the Royal Society of New Zealand. 4(3), 345-377.

[11] Gomes UL, Paragó C. 2001. Espécie nova de Rajídeo (Chondrichthyes, Rajiformes) Do Atlântico sul occidental. Boletim do Museu Nacional do Rio de Janeiro, Nova Série, Zoologia. 448, 1-10.

[12] Hulley PA. 1970. An investigation of the Rajidae of the west and south coasts of Southern Africa. Annals of the South African Museum. 55(4), 151-220.

[13] Hunter E, Buckley AA, Stewart C, Metcalfe JD. 2005. Migratory behavior of the thornback ray, *Raja clavata*, in the southern North Sea. Journal of the Marine Biological Association of the United Kingdom. 85, 1095-1105.

[14] Ishihara H. 1984. Second record of the rare skate *Anacanthobatis borneensis* form the East China Sea. Japanese Journal of Ichthyology. 30(4), 448-451.

[15] Ishihara H. 1987. Revision of the Western North Pacific species of the Genus Raja. Japanese Journal of Ichthyology. 34(3), 241-254.

[16] Ishihara H, Ishiyama R. 1985. Two new North Pacific skates (Rajidae) and a revised key to *Bathyraja* in the area. Japanese Journal of Ichthyology. 32(2), 143-179.

[17] Ishiyama R. 1952. Studies on the rays and skates belonging to the family Rajidae, found in Japan and adjacent regions. 4. A revision of three genera of Japanese rajids, with descriptions of one new genus and four new species mostly occured [sic] in northern Japan. The Journal of The Shimonoseki College of Fisheries, 2(2), 1-34, pls.1-4.

[18] Ishiyama R. 1958. Studies on the rajid fishes (Rajidae) found in the waters around Japan. Journal of the Shimonoseki College of Fisheries, 7 (2/3), 193–394, pls. 1-3.

[19] Ishiyama R, Ishihara H. 1977. Five new species of skates in the genus *Bathyraja* from the Western North Pacific, with reference to their interspecific relationships. Japanese Journal of Ichthyology. 24(2), 71-90.

[20] Jeong CH, Nakabo T. 1997. *Raja koreana*, a new species of skate (Elasmobranchii, Rajoidei) from Korea. Ichthyological Research. 44, 413-420.

[21] Jeong CH, Nakabo T. 2009. *Hongeo*, a new skate genus (Chondrichthyes: Rajidae), with description of the type species. Ichthyological Research. 56, 140-155.

[22] Krefft G. 1965. Die ichthyologische Ausbeute der ersten Westafrika-Fahrt des fischereitechnischen Forschungsschiffes "Walter Herwig". Raja herwigi spec. nov., eine neue Rochenart aus dem Seegebiet der Kapverden. 209-216.

[23] Last PR. 2008. New short-snout members of the skate genus *Dipturus* (Rajoidei: Rajidae) from Australian seas. Pp. 53-98. In: PR Last, WT White, JJ Pogonoski, DC Gledhill (eds) Descriptions of New Australian Skates. CSIRO Marine and Atmospheric Research Paper 021, 181 pp.

[24] Last PR, Alava M. 2013. *Dipturus amphispinus* sp. nov., a new longsnout skate (Rajoidei: Rajidae) from the Philippines. Zootaxa. 3752(1), 214-227.

[25] Last PR, Fahmi, Ishihara H. 2010. Okamejei cairae sp. nov. (Rajoidei: Rajidae), a new skate from the South China Sea. Pp. 89–100. In: PR Last, WT White, JJ Pogonoski (eds) Descriptions of New Sharks and Rays from Borneo. CSIRO Marine and Atmospheric Research Paper 032, 165 pp.

[26] Last PR, Gledhill DC. 2008. A new skate of the genus *Dentiraja* (Rajoidei: Rajidae) from southern Australia. Pp 109-118. In: PR Last, WT White, JJ Pogonoski, DC Gledhill (eds)

Descriptions of New Australian Skates. CSIRO Marine and Atmospheric Research Paper 021, 181 pp.

[27] Last PR, Gledhill DC. 2008. A new species of round skate, *Irolita westraliensis* sp. nov. (Rajoidei: Arhynchobatidae), from northern Western Australia. Pp 173-181. In: PR Last, WT White, JJ Pogonoski, DC Gledhill (eds) Descriptions of New Australian Skates. CSIRO Marine and Atmospheric Research Paper 021, 181 pp.

[28] Last PR, Gledhill DC. 2008. Two new skates of the genus *Okamejei* (Rajoidei: Rajidae) from the south-east Indian Ocean. Pp. 119-134. In: PR Last, WT White, JJ Pogonoski, DC Gledhill (eds) Descriptions of New Australian Skates. CSIRO Marine and Atmospheric Research Paper 021, 181 pp.

[29] Last PR, Mallick S, Yearsley GK. 2008. A review of the Australian skate genus *Pavoraja* Whitley (Rajiformes: Arhynchobatidae). Zootaxa. 1812, 1-45.

[30] Last PR, McEachran JD. 2006. New softnose skate genus *Brochiraja* from New Zealand (Rajidae: Arhynchobatinae) with description of four new species. New Zealand Journal of Marine and Freshwater Research. 40, 65-90.

[31] Last PR, McEachran JD. 2006. *Notoraja hirticauda*, a new species of skate (Chondrichthyes: Rajoidei) from the south-eastern Indian Ocean. Memoirs of Museum Victoria. 63(1), 65-75.

[32] Last PR, Séret B. 2008. Three new legskates of the genus *Sinobatis* (Rajoidei: Anacanthobatidae) from the Indo–West Pacific. Zootaxa. 1671, 33-58.

[33] Last PR, Séret B. 2012. Two new softnose skates of the genus *Brochiraja* (Rajoidei: Arhynchobatidae) from the deepwater slopes and banks of the Norfolk Ridge (South-West Pacific). Zootaxa. 3155, 47-64.

[34] Last PR, Séret B. 2016. A new Eastern Central Atlantic skate *Raja parva* sp. nov. (Rajoidei: Rajidae) belonging to the *Raja miraletus* species complex. Zootaxa. 4147(4), 477-489.

[35] Last PR, Stehmann MFW. 2008. *Rajella challengeri* sp. nov., a new deepwater skate from southern Australia. Pp. 135-144. In: PR Last, WT White, JJ Pogonoski, DC Gledhill (eds) Descriptions of New Australian Skates. CSIRO Marine and Atmospheric Research Paper 021, 181 pp.

[36] Last PR, Stehmann MFW, Séret B. 2008. *Leucoraja pristispina* sp. nov., a new deepwater skate from Western Australia. Pp. 145-154. In: PR Last, WT White, JJ Pogonoski, DC Gledhill (eds) Descriptions of New Australian Skates. CSIRO Marine and Atmospheric Research Paper 021, 181 pp.

[37] Last PR, Weigmann S, Dumale D. 2016. A new skate genus *Orbiraja* (Rajiformes: Rajidae) from the Indo-West Pacific. Zootaxa. 4184(1), 52-62.

[38] Last PR, White WT, Pogonoski JJ. 2008. New skates of the genus *Dipturus* (Rajoidei: Rajidae) form Australian Seas. Pp. 9-51. In: PR Last, WT White, JJ Pogonoski, DC Gledhill (eds) Descriptions of New Australian Skates. CSIRO Marine and Atmospheric Research Paper 021, 181 pp.

[39] Martinez CM. From images contributed to Martinez et al. 2016.

[40] Martinez CM, Kao BH, Sparks JS, Wainwright PC. Comparative materials accessed for this study.

[41] Martinez CM, Rohlf FJ, Frisk MG. 2016. Re-evaluation of batoid pectoral morphology reveals novel patterns of diversity among major lineages. Journal of Morphology. 227, 482-493.

[42] McEachran JD. 1977. Variation in *Raja garmani* and the status of *Raja lentiginosa* (Pisces: Rajidae). Bulletin of Marine Science. 27(3), 423-439.

[43] McEachran JD. 1982. Revision of the South American Skate Genus *Sympterygia* (Elasmobranchii: Rajiformes). Copeia. 4, 867-890.

[44] McEachran JD. 1983. Results of the research cruises of FRV “Walther Herwig” to South America. LXI: Revision of the South American skate genus *Psammobatis* Günther, 1870 (Elasmobranchii: Rajiformes, Rajidae). Archiv für Fischereiwissenschaft. 34(1), 23-80.

[45] McEachran JD, Compagno LJV. 1979. A further description of *Gurgesiella furvescens* with comments on the interrelationships of Gurgesiellidae and Pseudorajidae (Pisces, Rajoidei). Bulletin of Marine Science. 29(4), 530-553.

[46] McEachran JD, Compagno LJV. 1980. Results of the research cruises of FRV “Walther Herwig” to South America. LVI: A new species of Skate from the southwestern Atlantic, *Gurgesiella dorsalifera* sp. nov. (Chondrichthyes, Rajoidei). Archiv für Fischereiwissenschaft. 31(1), 1-14.

[47] McEachran JD, Last PR. 1994. New species of skate, *Notoraja ochroderma*, from off Queensland, Australia, with comments on the taxonomic limits of *Notoraja* (Chondrichthyes: Rajoidei). Copeia. 2, 413-421.

[48] McEachran JD, Last PR. 2008. New Deepwater skates of the genus *Notoraja* (Rajoidei: Arhynchobatidae) from southern Australia and the eastern Indian Ocean. Pp. 155-172. In: PR Last, WT White, JJ Pogonoski, DC Gledhill (eds) Descriptions of New Australian Skates. CSIRO Marine and Atmospheric Research Paper 021, 181 pp.

[49] Menni RC. 1973. Rajidae del litoral Bonaerense. I. Especies de los genereros *Raja*, *Bathyraja* y *Sympterygia* (Chondrichthyes). PHYSIS Sección A, Buenos Aires. 32(85), 413-439.

[50] Mnasri N, Boumaïza M, Ben-Amor MM, Capapé C. 2009. Polychromatism in the thornback ray, *Raja clavata* (Chondrichthyes:Rajidae) off northern Tunisian coast (central Mediterranean). Pan-American Journal of Aquatic Sciences. 4(4), 572-579

[51] Mnasri N, Boumaïza M, Capapé C. 2009. Morphological data, biological observations and occurrence of a rare skate, *Leucoraja circularis* (Chondrichthyes: Rajidae), off the northern coast of Tunisia (central Mediterranean). Pan-American Journal of Aquatic Sciences. 4(1), 70-78.

[52] Nozeres C. From images contributed to Martinez et al. 2016.

[53] Orlov AM. From images contributed to Martinez et al. 2016.

[54] Orlov AM, Cotton CF. 2013. New data on rare deepwater North Atlantic skate *Bathyraja pallida* (Forster, 1967) (Arhynchobatidae, Rajiformes). Journal of Ichthyology. 53(7), 465-477.

[55] Orr JW, Stevenson DE, Hoff GR, Spies I, McEachran JD. 2011. *Bathyraja panthera*, a new species of skate (Rajidae: Arhynchobatinae) from the western Aleutian Islands, and resurrection of the subgenus Arctoraja Ishiyama. NOAA Professional Paper, NMFS 11, 50 p.

[56] Raschi W, McEachran JD. 1991. *Rhinoraja longi*, a new species of skate from the outer Aleutian Islands, with comments on the status of *Rhinoraja* (Chondrichthyes, Rajoidei). Canadian Journal of Zoology. 69, 1889-1903.

[57] Séret B, Last PR. 2009. *Notoraja sapphira* sp. nov. (Rajoidei: Arhynchobatidae), a new deepwater skate from the slopes of the Norfolk Ridge (South-West Pacific). Zootaxa. 2153, 24-34.

[58] Stehmann M. 1976. *Breviraja caerulea* spec. nov. (Elasmobranchii, Batoidea, Rajidae); a new deep water skate and the first record of its genus in the North East Atlantic. Archiv für Fischereiwissenschaft. 27(2), 97-114.

[59] Stehmann M. 1976. Revision der Rajoiden-Arten des nördlichen Indischen Ozean und Indopazifik (Elasmobranchii, Batoidea, Rajiformes). Beaufortia. 24(315), 133–175.

[60] Stehmann M. 1978. *Raja "bathyphila"*, eine Doppelart des Subgenus *Rajella*: Wiederbeschreibung von *R. bathyphila* Holt & Byrne, 1908 und *Raja bigelowi* spec. nov. (Pisces, Rajiformes, Rajidae). Archiv für Fischereiwissenschaft. 29(1/2), 23-58.

[61] Stehmann M, Séret B. 1983. A new species of deep-water skate, Breviraja Africana sp. n. (Pisces, Batoidea, Rajidae), from the Eastern Central Atlantic Slope, and remarks on the taxonomic status of Breviraja Bigelow & Schroeder, 1948. Bulletin du Muséum national d'histoire naturelle. Section A, Zoologie, biologie et écologie animales, 5(3), 903-925.

[62] Stehmann MFW, Séret B, Costa EM, Baro J. 2008. Neoraja iberica n. sp., a new species of pygmy skate (Elasmobranchii, Rajidae) from the southern upper slope of the Iberian Peninsula (Eastern North Atlantic). Cybium. 32(1), 51-71.

[63] Stevenson DE. From images contributed to Martinez et al. 2016.

[64] Stevenson DE, Orr JW, Hoff GR, McEachran JD. 2004. *Bathyraja mariposa*: a new species of skate (Rajidae: Arhynchobatinae) from the Aleutian Islands. Copeia. 2, 305-314.

[65] Vargas-Caro C, Bustamante C, Bennett MB, Ovenden JR. 2017. Towards sustainable fishery management for skates in South America: The genetic population structure of *Zearaja chilensis* and *Dipturus trachyderma* (Chondrichthyes, Rajiformes) in the south-east Pacific Ocean. PLoS ONE 12(2), e0172255.

[66] Vargas-Caro C, Bustamante C, Lamilla J, Bennett MB. 2015. A review of longnose skates *Zearaja chilensis* and *Dipturus trachyderma* (Rajiformes: Rajidae). Universitas Scientiarum. 20(3), 321-359.

[67] Waite ER. 1909. Pisces. Part I. In: Scientific results of the New Zealand government trawling expedition, 1907. Records of the Canterbury Museum. 1(2), 131-155.

[68] Weigmann S, Stehman MFW, Thiel R. 2014. Complementary redescription of *Anacanthobatis ori* (Wallace, 1967) and its assignment to *Indobatis* n. g. (Elasmobranchii, Anacanthobatidae), with comments on other legskates. Zootaxa. 3779(2), 101-132.

[69] Weigmann S, Stehman MFW, Thiel R. 2015. *Okamejei ornata* n. sp., a new deep-water skate (Elasmobranchii, Rajidae) from the northwestern Indian Ocean off Socotra Islands. Deep-Sea Research II. 115, 18-29.

[70] Yearsly GK, Last PR. 1992. Pavoraja (Insentiraja) laxipella, a new subgenus and species of skate (Chondrichthyes: Rajoidei) from the western Pacific. Copeia. 3, 839-850.
