# Supplementary material for: Pectoral Dimorphism Is a Pervasive Feature of Skate Diversity and Offers Insight into their Evolution
Source: Integr Org Biol. 2019 Jun 15;1(1):obz012. doi: 10.1093/iob/obz012 (PMC7671108; doi:10.1093/iob/obz012)
Supplement: obz012_Supplementary_Data [file obz012_supplementary_data.zip › Figure S1.pdf]

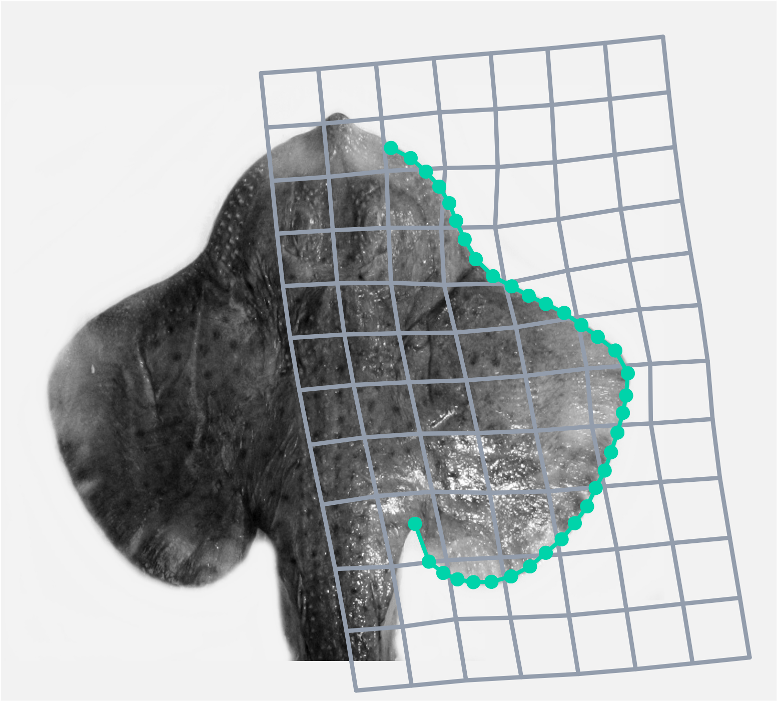

Figure S1: Landmarks used to capture pectoral fin outlines, shown with a warp grid relative to mean shape, overlaid on a male *Leucoraja erinacea*.
