# Supplementary material for: Pectoral Dimorphism Is a Pervasive Feature of Skate Diversity and Offers Insight into their Evolution
Source: Integr Org Biol. 2019 Jun 15;1(1):obz012. doi: 10.1093/iob/obz012 (PMC7671108; doi:10.1093/iob/obz012)
Supplement: obz012_Supplementary_Data [file obz012_supplementary_data.zip › Figure S2.pdf]

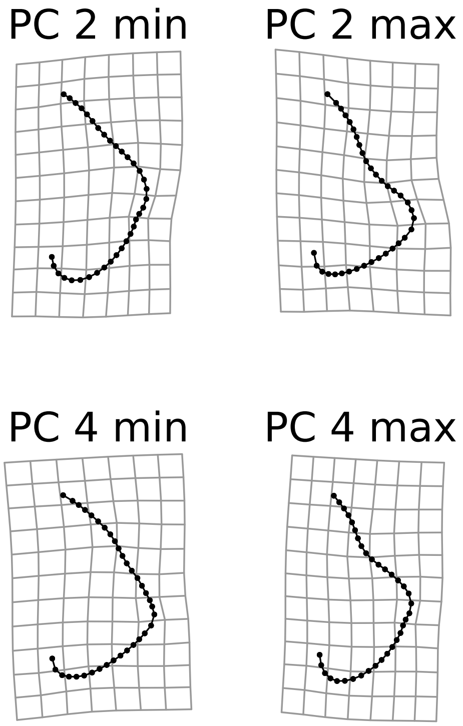

Figure S2: Deformation grids displaying shape change occurring on PCs 2 and 4 (accompanies manuscript figure 4) for pectoral fin data in 21 male-female pairs of skate genera and one guitarfish, *Zapteryx*.
