# Supplementary material for: Pectoral Dimorphism Is a Pervasive Feature of Skate Diversity and Offers Insight into their Evolution
Source: Integr Org Biol. 2019 Jun 15;1(1):obz012. doi: 10.1093/iob/obz012 (PMC7671108; doi:10.1093/iob/obz012)
Supplement: obz012_Supplementary_Data [file obz012_supplementary_data.zip › Figure S3.pdf]

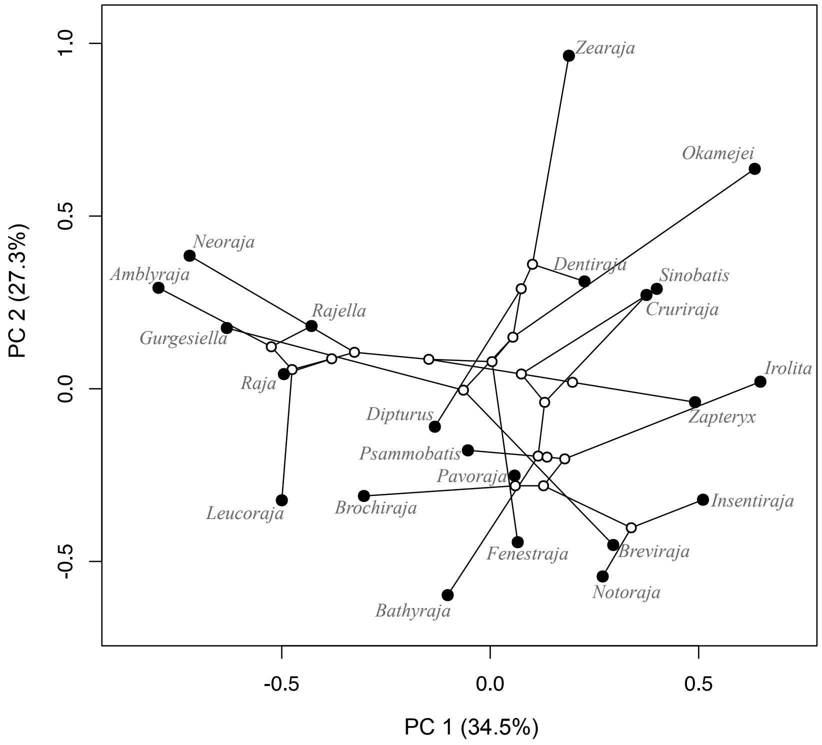

Figure S3: Phylomorphospace, containing PCs 1 and 2 of scaled dimorphism shape vectors in representatives from 21 genera of skate and one guitarfish (*Zapteryx*). Vectors were computed by subtracting female from male shape coordinates and then standardized by dividing by the Procrustes distance between the two. Ancestral nodes are shown as open circles and genera as filled circles.
