# Supplementary material for: Pectoral Dimorphism Is a Pervasive Feature of Skate Diversity and Offers Insight into their Evolution
Source: Integr Org Biol. 2019 Jun 15;1(1):obz012. doi: 10.1093/iob/obz012 (PMC7671108; doi:10.1093/iob/obz012)
Supplement: obz012_Supplementary_Data [file obz012_supplementary_data.zip › Figure S4.pdf]

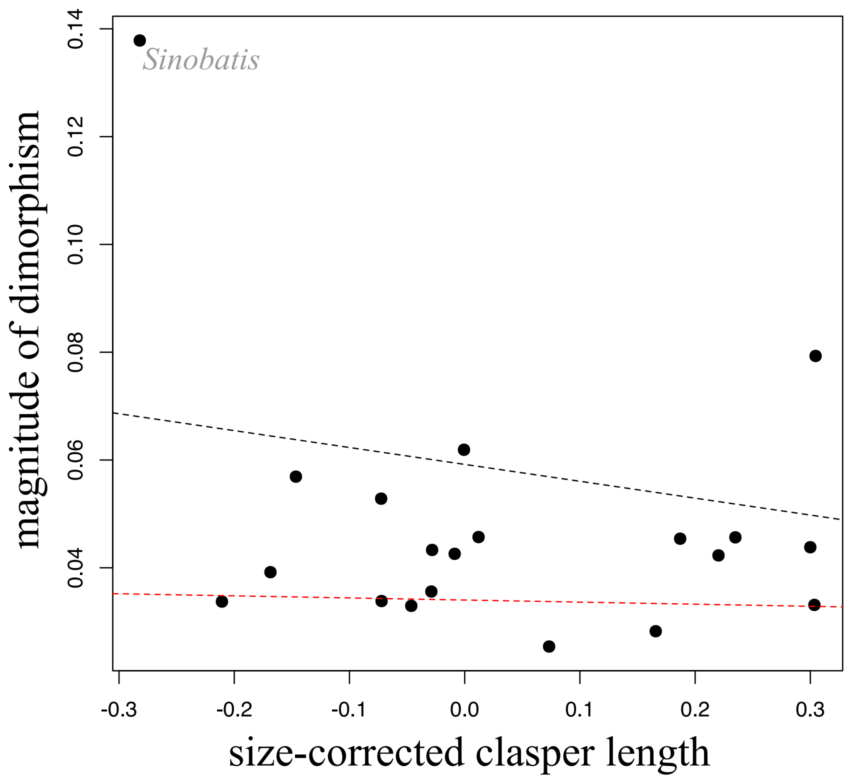

Figure S4: The magnitude of pectoral dimorphism (Procrustes distances between female and male shapes) in representatives from 20 genera of skate plotted against size-corrected clasper length. PGLS regression lines are shown for all genera (black line) and with the removal of an outlier, *Sinobatis* (red line).
