# Supplementary material for: Pectoral Dimorphism Is a Pervasive Feature of Skate Diversity and Offers Insight into their Evolution
Source: Integr Org Biol. 2019 Jun 15;1(1):obz012. doi: 10.1093/iob/obz012 (PMC7671108; doi:10.1093/iob/obz012)
Supplement: obz012_Supplementary_Data [file obz012_supplementary_data.zip › Table S1.pdf]

Table S1. List of skates positively identified as possessing sexually dimorphic pectoral fins. References are shown as numbers, matching full citations in Document S1. Determination of dimorphism was based on photographs (**P**), or from line drawings (**L**) from species' descriptions. Some species were determined to be provisionally dimorphic (*****), when only images of males were available. Species used for morphometric comparisons of male versus female pectoral shapes are also indicated by ⍋.

Amblyraja doellojuradoi [9,P]
Amblyraja georgiana [9,P]
Amblyraja hyperborea [8,P]
Amblyraja jenseni [54,P]
Amblyraja radiata [52,P,⍋]
Anacanthobatis americanus [3,9,L]
Arhynchobatis asperrimus [10,67,P]
Atlantoraja castelnaui [9,P]
Atlantoraja cyclophora [9,P]
Atlantoraja platana [9,P]
Bathyraja abyssicola [63,P]
Bathyraja brachyurops [9,49,P]
Bathyraja caeluronigricans [19,P]
Bathyraja cousseauae [9,P]
Bathyraja diplotaenia [17,P]
Bathyraja interrupta [63,P]
Bathyraja isotrachys [16,P]
Bathyraja lindbergi [19,P]
Bathyraja maculata [19,63,P]
Bathyraja mariposa [64,P,⍋]
Bathyraja matsubarai [17,P]
Bathyraja meridionalis [9,P*]
Bathyraja minispinosa [19,P]
Bathyraja notoroensis [19,P]
Bathyraja pallida [54,P]
Bathyraja panthera [55,P]
Bathyraja parmifera [55,P]
Bathyraja smirnovi [55,P]
Bathyraja smithii [9,P]
Bathyraja spinicauda [8,P]
Bathyraja taranetzi [56,P]
Bathyraja trachouros [17,L]
Bathyraja trachura [16,P]
Beringraja binoculata [63,P]
Breviraja spinosa [9,39,P,⍋]
Brochiraja albilabiata [30,P*]
Brochiraja asperula [10,L]
Brochiraja heuresa [33,P,⍋]
Brochiraja leviveneta [30,P*]
Brochiraja microspinifera [30,P*]
Brochiraja spinifera [10,L]
Cruriraja atlantis [2,L]
Cruriraja hulleyi [1,P,⍋]
Cruriraja rugosa [39,P]
Dentiraja flindersi [26,P,⍋]
Dipturus amphispinus [24,P]
Dipturus apricus [38,P]
Dipturus australis [9,23,P]
Dipturus canutus [23,P]
Dipturus confusus [23,P]
Dipturus endeavouri [23,P]
Dipturus falloargus [23,P,⍋]
Dipturus flavirostris [9,P*]
Dipturus grahami [23,P]
Dipturus healdi [38,P]
Dipturus innominatus [10,P]
Dipturus johannisdavesi [9,P*]
Dipturus kwangtungensis [15,P]
Dipturus laevis [8,39,P]
Dipturus mennii [11,P*]
Dipturus oculus [23,P]
Dipturus queenslandicus [38,P]
Dipturus trachydermus [66,P]
Fenestraja atripinna [40,P]
Fenestraja cubensis [40,P]
Fenestraja plutonia [40,P,⍋]
Fenestraja sibogae [59,P*]
Fenestraja sinusmexicanus [40,P]

Table S1. continued

Gurgesiella atlantica [9,39,P]	*Pavoraja umbrosa* [29,P]
Gurgesiella dorsalifera [46,P,⍋]	*Psammobatis bergi* [9,P]
Gurgesiella furvescens [45,P]	*Psammobatis extenta* [44,P]
Hongeo koreana [20,21,P]	*Psammobatis lentiginosa* [9,39,P]
Indobatis ori [68,P]	*Psammobatis normani* [44,P,⍋]
Insentiraja laxipella [70,P,⍋]	*Psammobatis rudis* [9,P]
Irolita westraliensis [27,P,⍋]	*Psammobatis rutrum* [9,P*]
Leucoraja circularis [9,51,P/L]	*Psammobatis scobina* [9,P*]
Leucoraja erinacea [39,P,⍋]	*Raja asterias* [6,P]
Leucoraja fullonica [9,P]	*Raja bahamensis* [4,P]
Leucoraja garmani [9,42,P/L]	*Raja brachyura* [5,6,P]
Leucoraja lentiginosa [9,42,P]	*Raja clavata* [9,13,50,P]
Leucoraja ocellata [39,P]	*Raja eglanteria* [39,P,⍋]
Leucoraja pristispina [36,P]	*Raja equatorialis* [9,P*]
Leucoraja wallacei [9,P]	*Raja herwigi* [22,P*]
Malacoraja senta [7,8,39,P]	*Raja miraletus* [9,34,P]
Neoraja africana [61,P]	*Raja montagui* [9,P*]
Neoraja caerulea [58,P]	*Raja radula* [9,P]
Neoraja iberica [62,P,⍋]	*Raja rhina* [63,P]
Notoraja azurea [48,P]	*Raja texana* [39,P]
Notoraja hirticauda [31,P]	*Raja velezi* [9,P]
Notoraja ochroderma [47,P]	*Rajella bathyphila* [60,P]
Notoraja sapphira [57,P,⍋]	*Rajella bigelowi* [60,P]
Notoraja sticta [48,P]	*Rajella caudaspinosa* [12,P]
Okamejei arafurensis [28,P,⍋]	*Rajella challengeri* [35,P,⍋]
Okamejei boesemani [15,P*]	*Rajella fyllae* [52,P]
Okamejei cairae [25,P]	*Rajella kukujevi* [53,P]
Okamejei hollandi [15,25,P]	*Rhinoraja longicauda* [17,P]
Okamejei kenojei [15,P]	*Rhinoraja macloviana* [9,P*]
Okamejei leptoura [28,P]	*Rhinoraja multispinis* [9,P]
Okamejei meerdervoortii [15,P]	*Rhinoraja odai* [18,P]
Okamejei ornata [69,P]	*Rioraja agassizii* [9,P]
Okamejei schmidti [18,L]	*Rostroraja alba* [7,9,P]
Orbiraja jensenae [9,37,P]	*Sinobatis borneensis* [14,P]
Orbiraja powelli [9,P*]	*Sinobatis bulbicauda* [32,P,⍋]
Pavoraja alleni [29,P⍋]	*Sinobatis filicauda* [32,P]
Pavoraja mosaica [29,P]	*Sympterygia acuta* [9,49,P]
Pavoraja nitida [29,P]	*Sympterygia bonapartii* [49,P]
Pavoraja pseudonitida [29,P]	*Sympterygia brevicaudata* [43,P]

**Table S1.** continued
Zearaja chilensis [65,P,4]
Zearaja nasuta [10,P]
