# Supplementary material for: Pectoral Dimorphism Is a Pervasive Feature of Skate Diversity and Offers Insight into their Evolution
Source: Integr Org Biol. 2019 Jun 15;1(1):obz012. doi: 10.1093/iob/obz012 (PMC7671108; doi:10.1093/iob/obz012)
Supplement: obz012_Supplementary_Data [file obz012_supplementary_data.zip › Table S2.pdf]

Table S2. Specimen information for *Fenestraja plutonia* , including sex, maturation state, and body size.

Catalog Number	Sex	Maturity	Disc Width (mm)
AMNH 76398	female	mature	93.19
AMNH 76398	male	mature	95.83
AMNH 76398	female	immature	83.23
AMNH 75988	female	mature	91.53
AMNH 75988	female	mature	97.41
AMNH 75988	male	immature	66.82
AMNH 76564	male	mature	107.94
AMNH 76564	female	mature	98.47
AMNH 75998	female	mature	88.03
AMNH 76398	female	immature	83.83
AMNH 76398	female	mature	94.14
AMNH 76398	male	mature	85.42
AMNH 84220	female	mature	100.25
AMNH 76398	male	mature	90.68
AMNH 76398	male	mature	87.23
AMNH 76398	male	mature	97.66
AMNH 76398	male	mature	93.44
AMNH 75988	male	immature	50.62
AMNH 75988	female	mature	92.12
AMNH 75998	male	mature	96.52
AMNH 76564	female	mature	108.03
MCZ 51827	female	mature	93.32
MCZ 51827	female	mature	95.26
MCZ 51827	male	mature	98.14
MCZ 51827	male	mature	99.68
MCZ 51827	male	mature	107.12
MCZ 51827	male	mature	107.70
MCZ 51827	female	mature	89.52
MCZ 36493	female	immature	71.52
MCZ 36493	male	immature	82.42
MCZ 36493	male	immature	75.50
MCZ 36493	male	immature	77.94
MCZ 36493	female	immature	55.55
MCZ 36493	female	immature	67.40
MCZ 36493	female	immature	76.98
MCZ 36493	female	immature	72.26
MCZ 36493	female	mature	92.25
MCZ 36493	female	mature	96.05
MCZ 36493	male	mature	103.50
MCZ 36493	male	mature	109.54
MCZ 36493	female	mature	92.01
MCZ 36493	male	immature	68.57
MCZ 36493	male	immature	64.38
